# The Construction of a Stream Service Application with DeepStream and Simple Realtime Server Using Containerization for Edge Computing

**DOI:** 10.3390/s25010259

**Published:** 2025-01-05

**Authors:** Wen-Chung Shih, Zheng-Yao Wang, Endah Kristiani, Yi-Jun Hsieh, Yuan-Hsin Sung, Chia-Hsin Li, Chao-Tung Yang

**Affiliations:** 1 Department of M-Commerce and Multimedia Applications, Asia University, Taichung City 413305, Taiwan; wjshih@asia.edu.tw; 2 Department of Computer Science, Tunghai University, Taichung City 407224, Taiwan; z.yao0103@gmail.com (Z.-Y.W.); endahkristi@gmail.com (E.K.); s08351014@thu.edu.tw (Y.-J.H.); s08351011@thu.edu.tw (Y.-H.S.); 3 Department of Informatics, Krida Wacana Christian University, Jakarta 11470, Indonesia; 4 iAMBITION TECHNOLOGY Co., Ltd., 3F., No. 159-1, Sec. 1, Zhongxing Rd., Dali District, Taichung City 412031, Taiwan; alanli8941@gmail.com; 5 Research Center for Smart Sustainable Circular Economy, Tunghai University, No. 1727, Sec. 4, Taiwan Boulevard, Taichung City 407224, Taiwan

**Keywords:** Docker, edge computing, simple realtime server, deepstream, Jetson Xavier NX

## Abstract

This paper addresses the increasing demand for efficient and scalable streaming service applications within the context of edge computing, utilizing NVIDIA Jetson Xavier NX hardware and Docker. The study evaluates the performance of DeepStream and Simple Realtime Server, demonstrating that containerized applications can achieve performance levels comparable to traditional physical machines. The results indicate that WebRTC provides superior low-latency capabilities, achieving delays of around 5 s, while HLS typically experiences delays exceeding 10 s. Performance tests reveal that CPU usage for WebRTC can exceed 40%, which is higher than that of HLS and RTMP, while memory usage remains relatively stable across different streaming protocols. Additionally, load testing shows that the system can support multiple simultaneous connections, but performance degrades significantly with more than three devices, highlighting the limitations of the current hardware setup. Overall, the findings contribute valuable insights into building efficient edge computing architectures that support real-time video processing and streaming.

## 1. Introduction

Edge computing is a distributed computing architecture that transfers applications, data storage, and computing tasks from centralized computing in the cloud to the end of the network or to nodes at the edge for processing [1]. The cloud’s processing tasks are primarily centralized in the cloud and distributed to the edge nodes to enhance efficiency and reduce latency. In addition, because edge nodes are closer to terminal users, data processing and transmission are faster, and network transmission is reduced. When the network connection is interrupted, part of the calculations or services handed over to the edge processing can continue to be completed, achieving high overall service availability [2]. Driven by artificial intelligence (AI) technologies (such as machine learning and deep neural networks), edge intelligence (EI) has been recognized as one of the main missing elements in 5G networks, and it is likely to become the critical factor in the realization of 5G networks [3].

The most common streaming video recognition function can also be built through the edge computing architecture [4]. In particular, large manufacturers such as NVIDIA have launched various hardware and software support for edge computing. The deep learning part is used in many fields, such as various learning frameworks for vision, speech, and NLP. NVIDIA provides extensive support at the core of the deep learning framework. Moreover, NVIDIA offers support for various software libraries such as cuDNN, DeepStream, TensorRT, and cuBLAS, which are compatible with a wide range of hardware. These packages and tools allow us to build our environment faster. Despite the abundance of convenient tools at our disposal, we may encounter specific challenges and issues during their integration. For instance, once we have successfully identified the development object, we typically connect the Edge device to the screen for confirmation, a process that can be quite troublesome. Therefore, this paper proposes an architecture that addresses the challenges we face when browsing streaming images on the Edge side, enabling users to directly view the operation results through the web page while creating their edge computing architecture [5,6].

This paper uses Docker and Docker Compose to integrate Deepstream and Simple Realtime Server environments and uses NVIDIA Jetson Xavier NX to build the entire system. This paper also provides detailed explanations for errors or usage skills encountered when using Deepstream on NVIDIA Jetson Xavier NX. In addition to the integrated environment, tests and comparisons are also made to use Deepstream and RTMP, WebRTC, HLS, and other streaming formats provided by Simple Realtime Server in Docker. This paper presents several significant findings and innovations related to the construction of a stream service application using DeepStream and Simple Realtime Server on NVIDIA Jetson Xavier NX hardware. Here are the key highlights:

- Performance Comparison: The study demonstrates that the performance and efficiency of the implementation in Docker are comparable to that of traditional physical machines. This finding is crucial as it validates the use of containerization for deploying applications without sacrificing performance.
- WebRTC Efficacy: The paper evaluates the efficacy of WebRTC as a streaming format, highlighting its low latency capabilities (around 5 s) compared to other formats like HLS, which typically experiences delays exceeding 10 s. This positions WebRTC as a favorable option for real-time communication applications.
- Cross-Platform Compatibility: The research emphasizes WebRTC’s robust cross-platform support, functioning effectively across various operating systems and devices, including Windows, macOS, Android, and iOS. This broad compatibility enhances the usability of the application in diverse environments.
- Load Testing Insights: The findings reveal that while the system can support multiple simultaneous connections, performance degrades significantly with more than three devices. This insight is critical for understanding the limitations of the current hardware setup and informs future scalability considerations.
- Integration of Technologies: The paper showcases the successful integration of multiple technologies, including DeepStream, Simple Realtime Server, and Docker, to create a cohesive streaming service. This integration not only simplifies deployment but also enhances the overall system’s performance and reliability.

These findings contribute to the growing body of knowledge in edge computing and real-time streaming applications, providing a foundation for future research and development in this area. The paper organizes its structure as follows: Section 2 provides a background review of the technologies utilized, including Docker, DeepStream, and various streaming protocols. Section 3 details the materials and methods employed in the experiments, including the system architecture and experimental environment. Section 4 presents the experimental results, comparing the performance of different streaming formats and the efficacy of the integrated system. Finally, Section 5 discusses the implications of the findings and outlines potential areas for future research.

## 2. Background Review and Related Study

We provide the background of this paper and information about several packages used, including Docker, DockerCompose, Simple Realtime Server, Apache, and various streaming formats.

### 2.1. Docker and Virtual Machines

Docker and virtual machines (VMs) share some similarities, but they also have key differences that set them apart [7]. Both technologies allow for the deployment of applications in isolated environments, which enhances security and resource management. However, Docker containers are generally lighter than VMs because they do not require a full operating system for each instance. Instead, containers share the host operating system’s kernel, which allows for faster startup times and reduced overhead.

In a virtual machine setup, a hypervisor is needed to allocate physical resources such as CPU and memory to each VM, creating a separate operating system environment for each instance [8]. This isolation can lead to higher resource consumption compared to Docker, where containers run directly on the host machine without the need for a hypervisor. As a result, Docker can be more efficient in terms of resource utilization, making it particularly suitable for microservices and cloud-native applications.

Docker is somewhat similar to a virtual machine, which is usually called a VM (Virtual Machine). A virtual machine is the virtual presentation or simulation environment of the relevant resources of the physical computer, enabling the creation of multiple virtual machines on a single physical machine; each virtual machine has its own operating system (OS) and applications. The virtual machine cannot directly interact with the physical computer. Instead, a lightweight software layer called the hypervisor is required to coordinate the processing between the virtual machine and the underlying physical hardware. The hypervisor is responsible for allocating the computing resources (such as processors, memory, and storage devices) of the physical machine to each virtual machine, and it allows each virtual machine to be isolated from each other without affecting each other. The application or environment running in Docker is called a container. Each container does not need to simulate an entire operating system; instead, it operates within the host machine’s operating system, utilizing the host’s kernel for resource management and isolation. This distinction is important because it highlights that while containers are lightweight and efficient, they still rely on the underlying operating system to function. Additionally, Docker’s architecture allows for multiple containers to run simultaneously on a single host, which enhances resource utilization compared to traditional virtual machines. Ali et al. [9] also show that it is very efficient and reliable to use in private and commercial clouds. Guidotti et al. explained in [10] that the use of Docker is a current trend in IT companies. Using Docker to deploy applications allows companies to design more competitive software products. At the same time, the use of Docker in related research can make the software reproducible, both in system development and research, which can be more convenient [11]. Docker Compose is faster and more convenient to use multiple containers. For example, in [12], Docker Compose is used to deploy a CaaS application. The use of Docker Compose to deploy applications on the Raspberry Pi, which is the same ARM architecture, is also worthy of reference [13].

### 2.2. DeepStream

DeepStream is a set of tools launched by NVIDIA. Now, more and more, we will need to use a web camera or a general USB camera for image recognition. The paper by Abdulghafoor [14] also used DeepStream. In order to allow users to have more complete and simple development tools, NVIDIA has integrated the DeepStream architecture.

**Figure 1:**
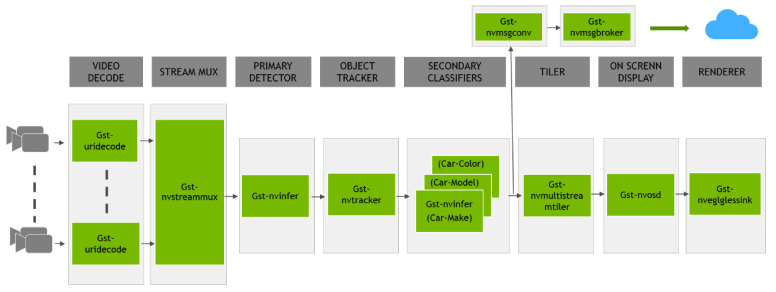
Deepstream architecture.

Figure 1 shows the architecture of the DeepStream framework. The architecture represents a video analytics pipeline using GStreamer plugins, commonly used in applications involving video processing and computer vision, such as traffic monitoring or surveillance. This pipeline efficiently processes video data, performs object detection and classification, and provides visualization and cloud connectivity for further actions or analysis.

- Video Decode, Gst-uridecode: Decodes video streams from multiple sources.
- Stream Mux, Gst-nvstreammux: Merges multiple video streams into a single stream for parallel processing.
- Primary Detector, Gst-nvinfer: Performs primary object detection using a neural network (e.g., detecting fire in the video).
- Object Tracker, Gst-nvtracker: Tracks detected objects across frames to maintain consistent object identities.
- Secondary Classifiers, Gst-nvinfer: Applies additional neural networks to classify attributes of detected objects, such as car color, model, and make.
- Tiler, Gst-nvmultistreamtiler: Arranges multiple video streams or frames in a tiled display, facilitating simultaneous viewing.
- On-Screen Display, Gst-nvosd: Renders metadata such as bounding boxes and labels onto the video frames for visualization.
- Renderer, Gst-nveglglessink: Outputs the processed video to a display or file.
- Cloud Integration:
  – Gst-nvmsgconv: Converts metadata into a format suitable for messaging.
  – Gst-nvmsgbroker**: Sends processed metadata to a cloud service for further analysis or storage.

### 2.3. Simple Realtime Server

Simple Realtime Server is a project on GitHub [15] which provides a simple and high-performance streaming server that supports RTMP/WebRTC/HLS/HTTP-FLV/SRT/GB28181. There are a variety of formats, among which the WebRTC streaming format provided in version 4.0 has a very real-time streaming screen output; it is therefore the final selected streaming protocol in this paper.

### 2.4. RTMP

RTMP (Real-Time Messaging Protocol) is a proprietary protocol originally developed by Macromedia for the transmission of streaming audio, film, and data between a Flash player and a server via the Internet. Macromedia was later acquired by Adobe Systems; this led to the release of an incomplete specification for public use. It has many variants [16]:

- The plain protocol using TCP port 1935 by default.
- RTMPS, transmitted through a TLS/SSL connection.
- RTMPE, encrypted with Adobe’s own security mechanism. Although the implementation details are proprietary, the mechanism uses industry-standard cryptographic primitives.
- RTMPT, encapsulated with HTTP to penetrate the firewall. RTMPT usually uses clear text requests on TCP ports 80 and 443 to bypass most corporate traffic filtering. Encapsulated conversations may carry pure RTMP, RTMPS or RTMPE packets.
- RTMFP, using UDP instead of TCP, instead of RTMP Chunk Stream. Adobe Systems has developed a secure real-time media streaming protocol suite that allows end users to directly connect to each other (P2P). The RTMP protocol (Real-Time Messaging Protocol) is used to transmit objects, video, and audio between Flash and the server. This protocol is based on the TCP protocol or the polling HTTP protocol. The RTMP protocol is like a container for storing data packets. These data can be either video/audio data in FLV or data in AMF format. A single connection can transmit multiple network streams through different channels, and the sizes of the packets passing through the channels are all fixed.

### 2.5. HLS

HTTP Live Streaming (abbreviated as HLS) is an HTTP-based streaming media network transmission protocol proposed by Apple; in a paper [17] by Durak, there is a comparison and analysis of HLS. The working principle is to first cut the entire media stream into small pieces of HTTP files and only download a small part each time it is downloaded. When the media stream is playing, the client can choose from many different alternative sources to download the same resource and download them at different rates, allowing the streaming media session to adapt to different data rates. When starting a streaming media session, to find available media streams, the client downloads an extended M3U (m3u8) playlist file containing metadata [18].

HLS only requests basic HTTP messages. Unlike RTMP, HLS can pass through any firewall or proxy server that allows HTTP data to pass through. It is also easy to use the content distribution network to transmit media streams.

### 2.6. WebRTC

Both HLS and RTMP has its own advantages and disadvantages. The SRS (Simple Realtime Server) used in this project is the core streaming platform, unlike traditional HLS and RTMP. Another emerging streaming format is WebRTC. WebRTC is the abbreviation of Web Real-Time Communication, which allows different users on the browser to perform peer-to-peer voice and video calls and data transmission without installing a browser plugin. Up to now, more and more browsers have joined the ranks of supporting WebRTC, including Apple’s Safari and Microsoft’s Edge. In addition, due to the characteristics of its open source and the vigorous development of related communities, many companies have used the WebRTC platform to customize their corporate communication and social communication applications [19].

WebRTC uses point-to-point UDP to transmit streaming data, so it has the advantage of close to ultra-low latency (real-time), but since each peer may be located under a firewall or NAT structure, it needs a set that can penetrate NAT. To coordinate and ensure a certain degree of network penetration between endpoints, WebRTC currently uses ICE, which is a combination of STUN and TURN, and then chooses a relatively suitable protocol according to different environments. Among them, STUN can be simply thought of as a UDP hole punching mechanism for NAT, and TURN is a relay server mechanism for multimedia streaming, mainly as a backup mechanism when the current one fails. But even with the above protocols, WebRTC still cannot meet 100% network penetration, which is worse than the general HTTP protocol (ex HLS), and it also lacks CDN support [20].

WebRTC has demonstrated robust cross-platform support, functioning effectively on major operating systems such as Windows, macOS, Android, and iOS. According to the document, supported browsers include Google Chrome, Mozilla Firefox, Microsoft Edge, and Safari, which are compatible with WebRTC on both desktop and mobile devices. This wide compatibility allows users to engage in real-time communication regardless of their device, enhancing the user experience. In addition to this, iOS 11, Google Chrome OS, Firefox OS, etc., are all supported. The actual test equipment is shown in Table 1. Tests on several common platforms found that Chrome, Firefox, and Safari can watch streaming images, regardless of the device on which they are used.

Despite its strengths, there are limitations to WebRTC’s functionality that need to be addressed [21]:

- Network Penetration Issues: WebRTC relies on protocols like ICE, STUN, and TURN to facilitate connections through firewalls and NATs. However, it does not guarantee 100% network penetration, which can lead to connectivity issues in certain environments.
- Performance Variability: While WebRTC offers low latency, the performance can vary significantly based on the device and network conditions. For instance, the aforementioned document notes that while WebRTC can achieve a delay of around 5 s, this can increase under suboptimal conditions, particularly when multiple devices are connected simultaneously.
- Resource Usage: WebRTC’s real-time capabilities demand higher CPU usage compared to other streaming protocols like HLS and RTMP. This can be a concern for devices with limited processing power, as the CPU usage can exceed 40% during operation, potentially affecting overall system performance.

### 2.7. YOLOv4

YOLO is a one-stage object detection method; that is, the input image file is taken as a whole. It only needs to perform a convolutional neural network architecture on this image file to determine the position and type of objects in the image, thus improving the recognition speed [22].

It is characterized by fast recognition speed. While ensuring the speed, a series of optimizations and improvements have been made to YOLOv3, which has greatly improved the accuracy of YOLOv4 version, reduced the use of hardware requirements, and enabled the development to approach maturity.

### 2.8. Edge Device

The NVIDIA Jetson Xavier NX demonstrates impressive performance for edge computing applications across various domains. It outperforms other Jetson platforms like the Nano and TX1 in processing complex data types such as 3D point clouds and hyperspectral images [23]. The Xavier NX does better than the Jetson Nano and the Raspberry Pi 4B with Intel Neural Compute Stick 2 at real-time object detection tasks using YOLO models [24,25]. The device also proves capable of running computationally intensive metagenomic analysis workflows efficiently, supporting fully offline operations in remote locations [26]. However, researchers observe that many tools lack optimization for edge devices, which presents opportunities for future development to fully utilize the Xavier NX’s capabilities [26].

### 2.9. Open Broadcaster Software

The use of Open Broadcaster Software (OBS) is justified in this system for several reasons. Firstly, OBS is a free and open-source software suite that supports recording and webcasting, making it accessible across multiple operating systems, including Windows, macOS, and Linux. This broad compatibility allows users to utilize OBS regardless of their preferred platform, enhancing usability. Secondly, OBS provides rich functionality, including real-time source and device capture, scene composition, encoding, recording, and broadcasting without limitations on upload traffic, resolution, or frame rate (FPS). This versatility is crucial for applications requiring high-quality video output, thereby improving the overall user experience. Additionally, OBS is integrated into the system to stream stable signals for identification purposes, as demonstrated with live video examples from Taitung Duoliang Station and Hualian Chike Mountain. This integration allows for the effective monitoring and management of live video streams, contributing to a seamless user experience when accessing real-time data through the web interface.

It is rich in functions and easy to operate, providing real-time source and device capture, scene composition, encoding, recording, and broadcasting, without limiting upload traffic, resolution, and frame rate (FPS, frame per second), and is mainly transmitted through the instant messaging protocol (RTMP, Real-Time Messaging Protocol), so it can support multiple live streaming platforms such as YouTube, Twitch.tv, Instagram, and Facebook [27]. Overall, the combination of OBS’s functionality, accessibility, and integration capabilities makes it an essential component of the streaming service architecture described in the paper.

### 2.10. Related Works

Singh et al. [28] published a container-as-a-service approach applied to processing applications using edge computing, and also implemented this using Docker. Although, in this paper, container service integration and migration solutions are used to achieve load balancing of system performance, and for some IoT applications that require low latency or higher transmission rates to improve performance, a software-defined edge computing is proposed, a lightweight and energy-efficient container-as-a-service (CaaS) approach to provide workloads for low-latency IoT applications is also present. It is also worth referring to and building similar CaaS functions.

Potdar et al. [7] also mentioned that server virtualization is a technological innovation widely used in IT companies. Virtualization provides a platform for different services to run operating systems on the cloud. It helps to build multiple virtual machines on a single basic physical computer in the form of a hypervisor or container. To host many microservice applications, emerging technologies introduce a model that consists of different operations performed by a smaller, single deployed service. Therefore, the demand for low-overhead virtualization technology is rapidly evolving. There are many lightweight virtualization technologies. Docker is one of them; it is an open source platform. This technology allows developers and system administrators to use the Docker engine to build, create, and run applications. In this paper, standard benchmark testing tools such as Sysbench, Phoronix, and Apache benchmarks are used to evaluate the performance of Docker containers and virtual machines, including CPU performance, memory throughput, storage read and write performance, load testing, and operating speed measurement.

In the work by Xu [29], it is mentioned that with the development of 5G technology, edge computing will place the computing center near the edge of the network with low latency, high security, and lightweight functions. The development of edge computing has also put forward a series of requirements for security, standardization, and uniformity. Docker container technology is lightweight, standardized, convenient, and securely isolated, so it can easily meet the existing needs of edge computing. The content mainly analyzes the vulnerability of Docker containers and summarizes the security issues faced by the application of Docker container technology in edge computing systems. It also introduces the container monitoring software Prometheus, and proposes a feasible edge computing risk monitoring model based on the Docker engine and Prometheus monitoring software. It also helps us to understand the security issues that Docker container technology has to face under edge computing systems.

Jansen [30] and H.S [31] have built complex systems through Docker; however, in addition to using Docker, it is also necessary to enable the container to be used efficiently. Brogi [32] discussed the benefits that can be gained by supporting the enhanced description of multi-container Docker applications. They also explain how to model such applications naturally in TOSCA, and how this allows automated management and reduces the time and cost required to develop such applications. Nguyen also mentioned in [33] how another mechanism to ensure that the files related to the VM/container management system remains in the cache of the host operating system. The results show that using these two technologies can speed up the startup duration of the VM by 2–13 times and the startup duration of the container by 2 times.

Deepika Saxena in [34] also mentioned that in the current situation, technological development is very different, and this difference lies in different platforms. Containers help to improve operational efficiency, version control, developer productivity, and environmental consistency. In this paper, we analyze the performance of Docker by using different applications or tools in a cloud environment.

Morabito et al. [35] evaluates container-based solutions for Internet of Things (IoT) service provisioning, focusing on two frameworks: the Container-Based Pair-Oriented IoT Service (CPIS) and the Container-Based Edge-Managed Clustering (CEMC) approach. The authors highlight the challenges posed by the increasing number of heterogeneous IoT devices and the need for lightweight virtualization technologies, such as Docker, to efficiently manage resources in constrained environments. They conducted a performance evaluation using a real IoT testbed, specifically utilizing Raspberry Pi devices to assess power consumption and resource usage during various tasks. The findings indicate that while the CEMC approach introduces some management overhead, it significantly enhances scalability and manageability for integrated IoT applications compared to the direct interactions of the CPIS approach.

Qiao et al. [36] introduces EdgeOptimize, a programmable containerized scheduler designed for managing time-critical tasks in Kubernetes (K8s)-based edge-cloud clusters, addressing the growing need for efficient service orchestration and request dispatching in edge computing environments. The authors highlight the significance of edge-cloud collaboration in meeting the low-latency requirements of IoT applications, emphasizing that traditional cloud computing models are insufficient for handling the demands of delay-sensitive services. The paper also compares EdgeOptimize with existing simulators and optimizers, demonstrating its advantages in fast model customization and low I/O blocking while being open-source. Experimental evaluations using real-world datasets show that the D3QN algorithm implemented in EdgeOptimizer outperforms other algorithms, such as the modified DDPG and K8s-native policies, in terms of request execution success rates. The findings suggest that EdgeOptimizer can effectively enhance the performance of time-critical tasks in edge-cloud collaboration scenarios.

Oleghe [37] discusses the challenges and methodologies related to container placement and migration in edge computing. It highlights the increasing demand for edge computing due to the growth of connected devices and the need for low-latency processing, predicting that by 2025, 75% of data will be processed outside traditional data centers. The paper emphasizes the role of container orchestration, particularly the scheduling algorithms that manage the allocation of computing requests to containers on heterogeneous edge nodes, which is described as an NP-hard problem. Various frameworks and algorithms are explored, including optimization models, multi-dimensional knapsack problems, and Markov Decision Processes, which help in formulating the container placement problem. The study also notes the importance of heuristic algorithms for achieving near-optimal solutions quickly, as well as the need for decentralized scheduling systems to handle the increasing complexity of edge computing tasks. Overall, the research aims to provide insights into effective scheduling models that can adapt to the dynamic nature of edge computing environments.

## 3. Materials and Methods

### 3.1. System Architecture

**Figure 2:**
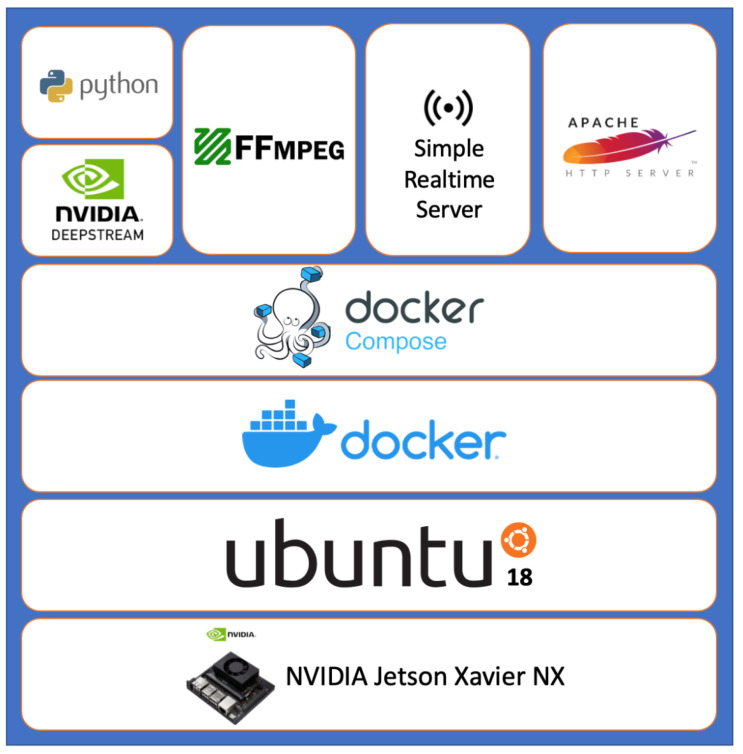
System architecture diagram.

The architecture proposed in this paper is shown in Figure 2. The hardware is NVIDIA Jetson Xavier NX (NVIDIA, Santa Clara, CA, USA), and the operating system is the SD Image provided by NVIDIA built-in Ubuntu 18.04. Additionally, we have installed Docker and Docker Composer, and have created multiple containers through Docker Composer management to integrate our services. In the related works [38] by List and Pentyala [39], they also use this method to build their services.

The keypoints of the architecture are listed as follows:

- Edge Computing: By running the entire pipeline on the Jetson Xavier NX, the system can perform real-time analysis without relying on a cloud-based infrastructure. This reduces latency and improves privacy.
- Containerization: Using Docker and Docker Compose simplifies the deployment and management of the application on the Jetson Xavier NX.
- Efficient Video Processing: DeepStream is specifically designed for efficient video processing on NVIDIA hardware, making it well-suited for this type of application.

### 3.2. Experimental Environment

**Figure 3:**
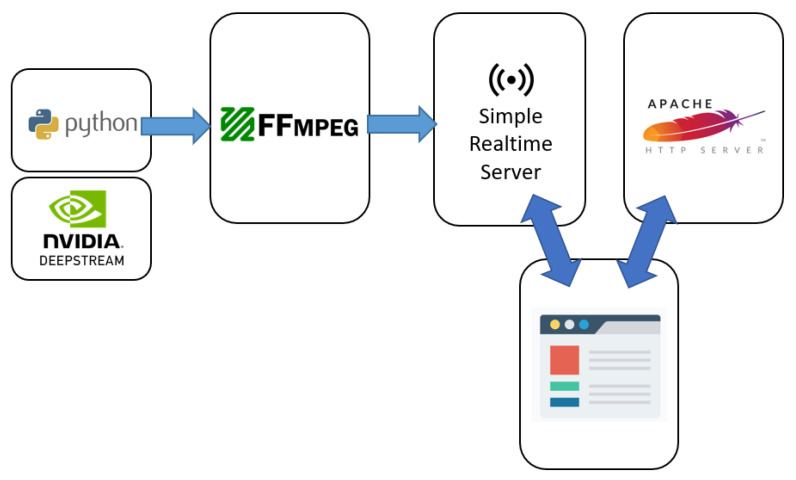
Container execution process.

This paper chooses NVIDIA’s Jetson Xavier NX hardware. NVIDIA’s Jetson Xavier NX can provide up to 21 trillion operations, suitable for use in embedded and edge systems. In addition to supporting high-performance computing and artificial intelligence, it can also handle video encoding and decoding functions. This paper uses Docker Compose to integrate deepstream, ffmpeg, Simple Stream Server, and Apache environment. The container execution process in Figure 3 allows for efficient and real-time video analysis, enabling applications like surveillance systems, video analytics, and interactive video experiences. The steps of the process are listed as follows.

- FFmpeg captures video from a source (camera, file) and encodes it into a suitable format.
- The video stream is sent to the Simple Realtime Server, which passes it to the DeepStream pipeline.
- DeepStream processes the video frames, performing object detection and face recognition using pre-trained models.
- The results from DeepStream are processed further on the server, potentially generating additional information or annotations.
- The processed video stream and any additional information are sent to the Apache HTTP server.
- The web browser receives the data from the server and displays the processed video stream with any overlays or annotations.

The detailed technical specifications provided by NVIDIA are shown in Table 2. The hardware mainly has 384 NVIDIA CUDA cores, 48 Tensor cores, 6 Carmel ARM CPUs, and 2 NVIDIA Deep Learning Accelerator (NVDLA) engines. The detailed software configuration of Jetson Xavier NX used in this paper is shown in Table 3, which includes all installed services in Docker Compose.

## 4. Experimental Results

In this section, we describe the performance comparison results, including Deepstream Efficacy, Simple Realtime Server Efficacy, and WebRTC Cross Platform Support.

### 4.1. Deepstream Efficacy

**Figure 4:**
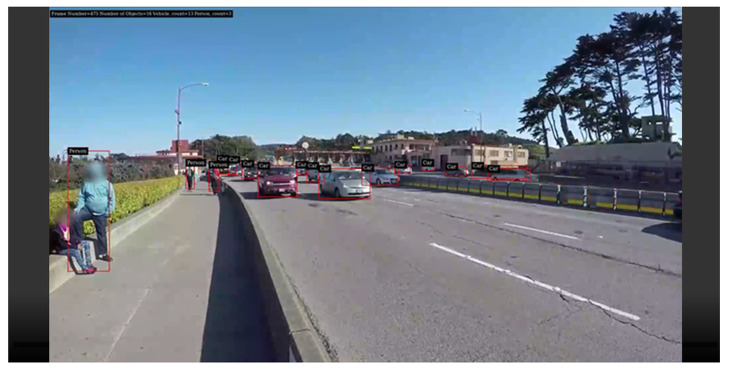
Web results screen.

After the container is successfully executed, we can open the web page directly to check the identified streaming screen as shown in Figure 4. We can also confirm the execution status of each container through docker ps -a. Due to the management through Docker Compose, when the container terminates unexpectedly, Docker Compose will automatically restart the container to avoid service interruption.

**Figure 5:**
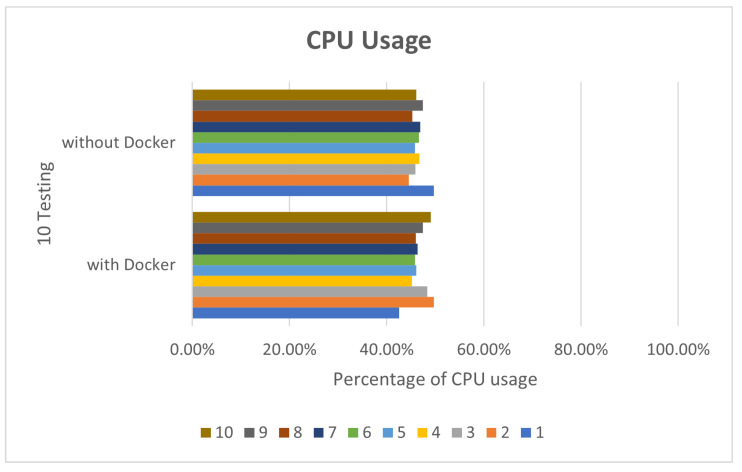
deepstream_test1 CPU usage.

**Figure 6:**
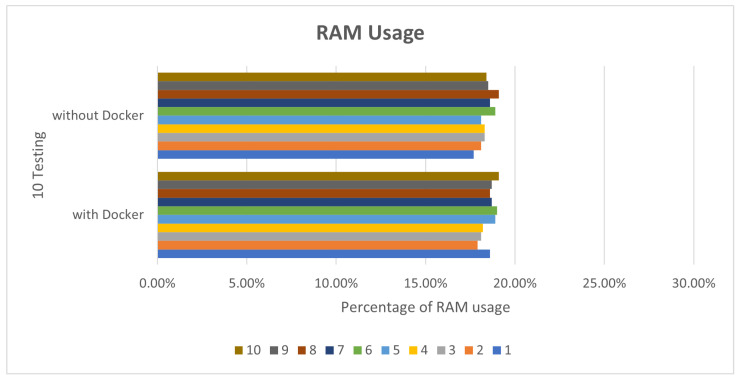
deepstream_test1 RAM usage.

In view of the performance, because Docker does not require Hypervisor to implement hardware resource virtualization, the programs executed on the Docker container directly use the hardware resources of the physical machine, so no matter what it is running, there is not much difference between Deepstream’s sample programs and the execution of Simple Realtime Server. In this paper, Deepstream provides sample programs by default for performance comparison. In the deepstream_test1 sample program, we perform 10 tests, respectively. The CPU and RAM usage are as shown in Figure 5 and Figure 6.

**Figure 7:**
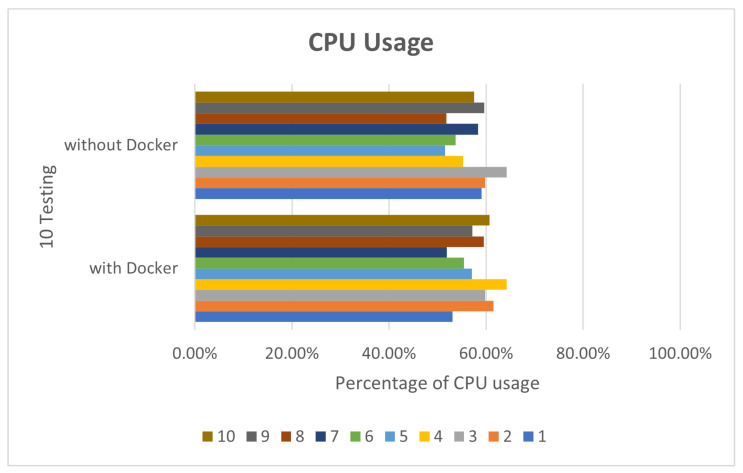
deepstream_test2 CPU usage.

**Figure 8:**
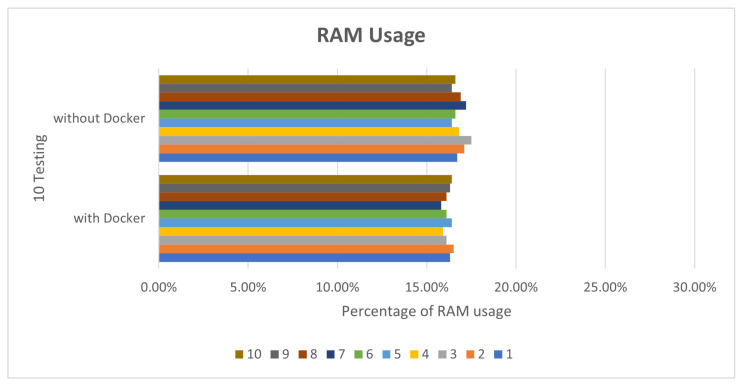
deepstream_test2 RAM usage.

In deepstream_test2, 10 tests were also performed. The CPU usage rate was about 42% to 49%, as shown in Figure 7. The memory part was not much different, about 17% to 18%, as shown in Figure 8.

**Figure 9:**
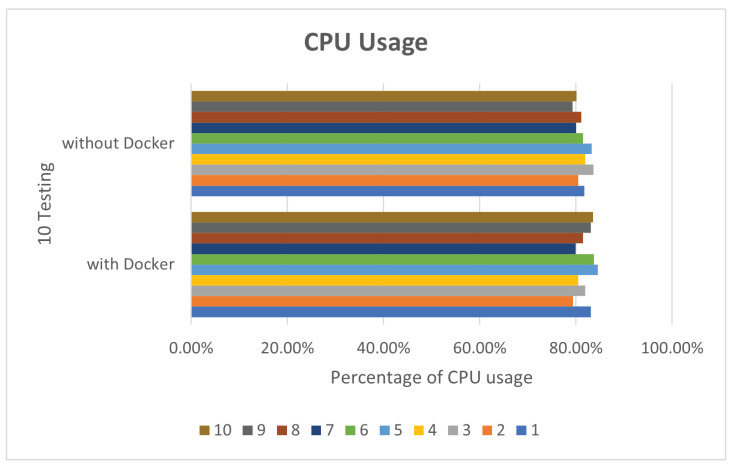
deepstream_test3 CPU usage.

**Figure 10:**
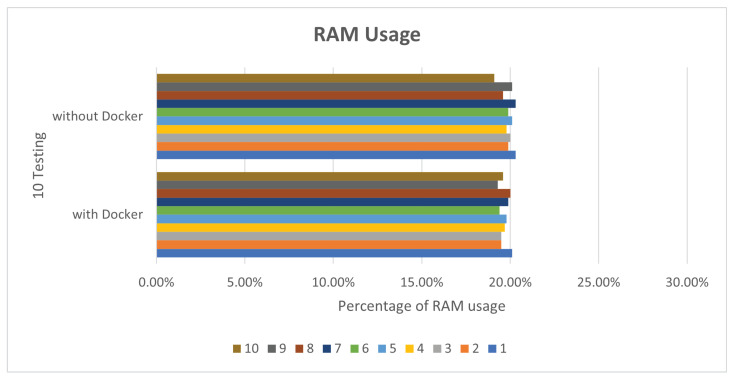
deepstream_test3 RAM usage.

**Figure 11:**
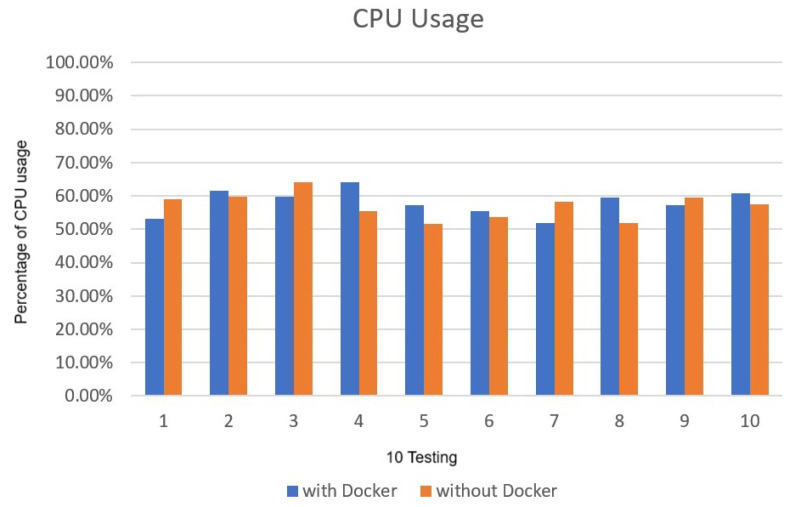
Comparison of average CPU usage.

**Figure 12:**
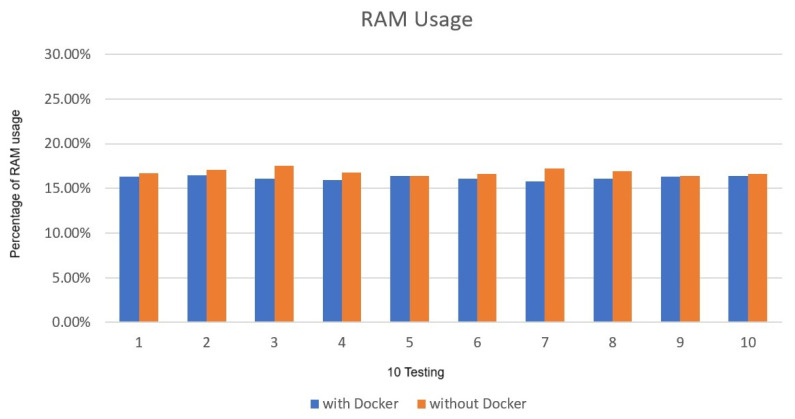
Comparison of average RAM usage.

The CPU and memory usage did not cause a large amount of usage due to the use of Docker. The CPU usage rate is about 79% to 83% in Figure 9, and the memory part is not much different, about 19% to 20% in Figure 10. The experimental results reveal that the CPU and RAM requirements are similar when executing Deepstream in Docker compared to without Docker. It can be proved that the effectiveness and efficiency of the implementation in Docker is no different from the general physical machine, as shown in Figure 11 and Figure 12.

### 4.2. Simple Realtime Server Efficacy

**Figure 13:**
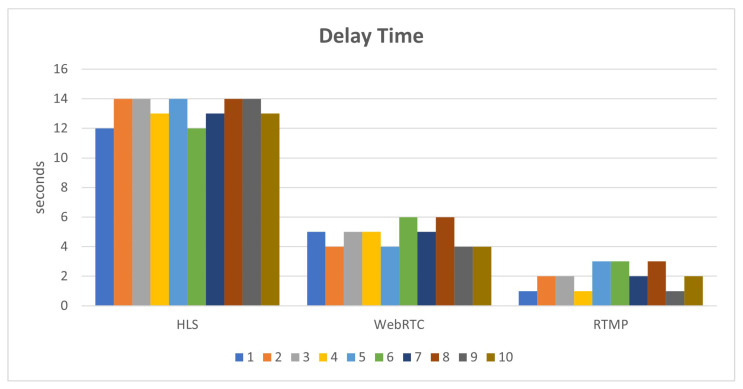
Simple Realtime Server efficacy.

This paper uses the Simple Realtime Server to compare the streaming formats WebRTC and HLS displayed on the web page. The results of the 10 tests for FPS output and film delay are shown in Figure 13. It can be found from the results that there will be better low-latency images in WebRTC format on web pages.

The basic principle of HLS is to correspondingly encode the video and audio media files to be streamed into a series of video and audio streaming fragments, usually a new ts file, and the server will create an m3u8 index file as the HLS playlist. When the player gets the live broadcast, it will parse the m3u8 index file to obtain the latest ts video clips one by one to play, thereby ensuring that users can see newer content whenever they connect. Compared with other live broadcast protocols, such as RTMP, RTSP, etc., the biggest difference in HLS is that the live broadcast terminal obtains continuous, short-duration media files instead of a complete data stream. The client continues to download and play these small files to achieve a live broadcast experience. The minimum delay of this theory is the length of a ts file, usually the length of 2–3 ts files. The general HLS segmentation strategy is 10 s per segment, which shows the shortcomings of HLS: common HLS live broadcast delays can reach 20–30 s, and high latency is unacceptable for live broadcasts that require instant interactive experience. HTTP is established on TCP, while HLS is established on short connection HTTP, which means that HLS needs to continuously establish a connection with the server. Each time TCP establishes a connection, the four waves that occur when it is disconnected will cause delay. However, HLS also has the following advantage: there is no need to consider firewall or proxy issues when using HLS because the data are transmitted through the HTTP protocol.

**Figure 14:**
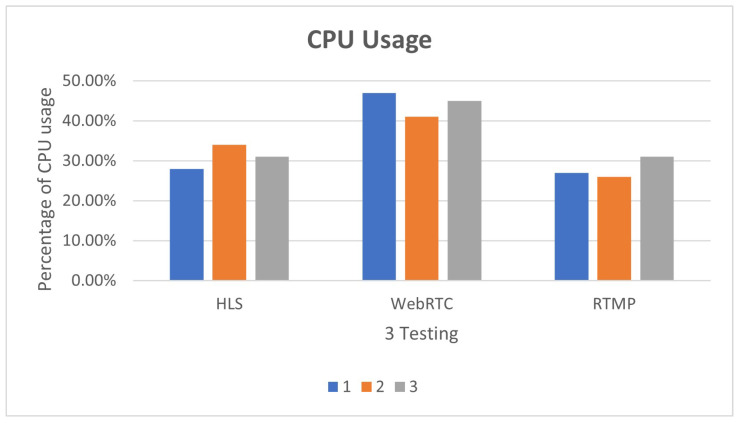
Different streaming protocols CPU usage.

**Figure 15:**
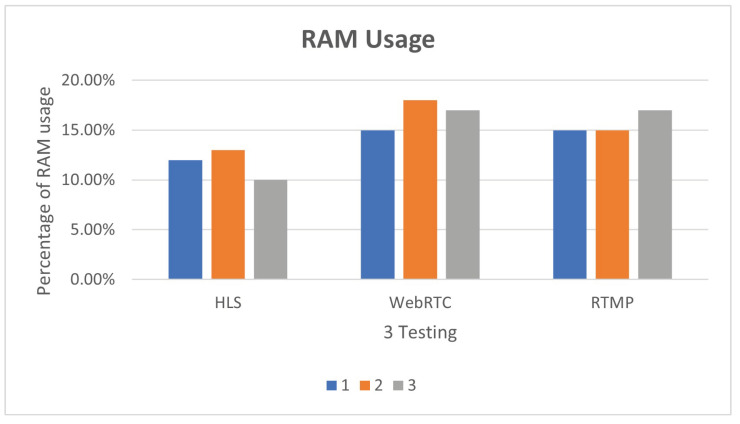
Different streaming protocols RAM usage.

The results of the first three tests are shown in Figure 14. Although there is less delay in using WebRTC, the CPU usage rate is higher than that of HLS. The reason may lie in the fact that WebRTC uses point-to-point links when establishing links, which can decode pictures and sounds in real-time. The requirements are relatively high. From the experimental results, it is obvious that in the three experiments, WebRTC occupies more than 40% of the CPU, which is higher than that of HLS and RTMP, but there is no obvious change in memory. The results of the first three tests are shown in Figure 15.

**Figure 16:**
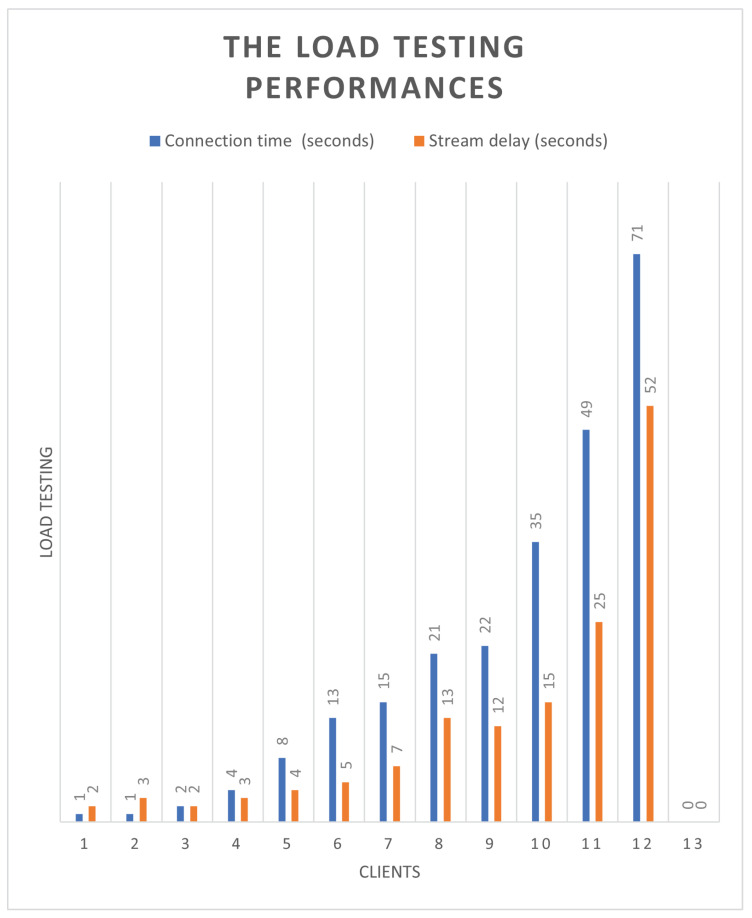
Load testing.

**Figure 17:**
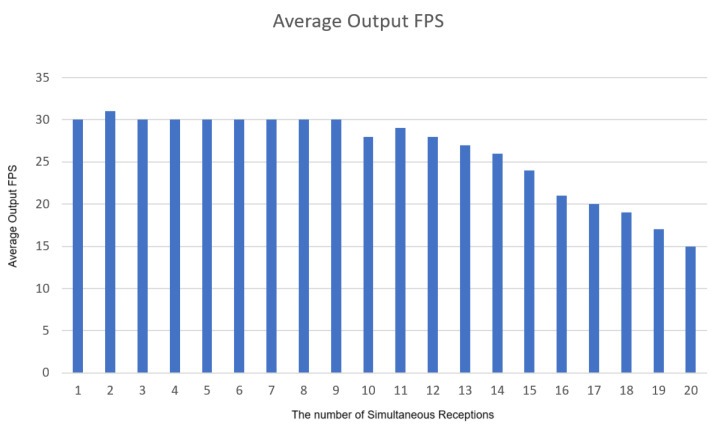
Average Output FPS.

Finally, for the load test shown in Figure 16, we used multiple devices to connect to the Simple Realtime Server at the same time to watch real-time video. It was found that more than three devices were connected at the same time, there was a significant delay time, and the connection time with the server became much longer. After more than 12 devices are connected, the connection cannot be reached; the server’s connection limit has been reached. From the experimental results, it is found that in the receiving signal source part, the number can reach 15 simultaneous receptions, and the FPS is stable, as shown in Figure 17.

## 5. Discussion

### 5.1. Image Recognition and Training

This subsection shows that the system built the YOLOv4 image recognition environment on Ubuntu 18.04.6LTS (Long Term Support), and trains the data to be recognized. The summary of Simple Realtime Server (SRS) is described in Table 4.

**Figure 18:**
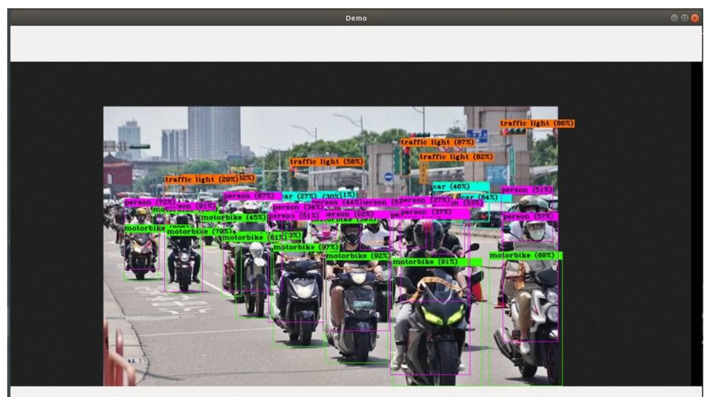
Vehicles identification training.

The inference is shown in Figure 18.

The summary of speed and accuracy from inference result is described in Table 5. It can be seen that each object has its own accuracy, with the speed of inference presented as FPS.

### 5.2. Export and Identify the Stream Signal

**Figure 19:**
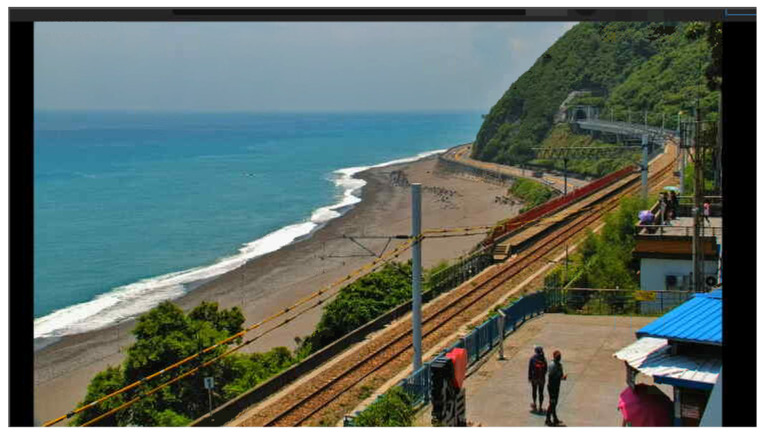
OBS (Open Broadcaster Software) window settings.

**Figure 20:**
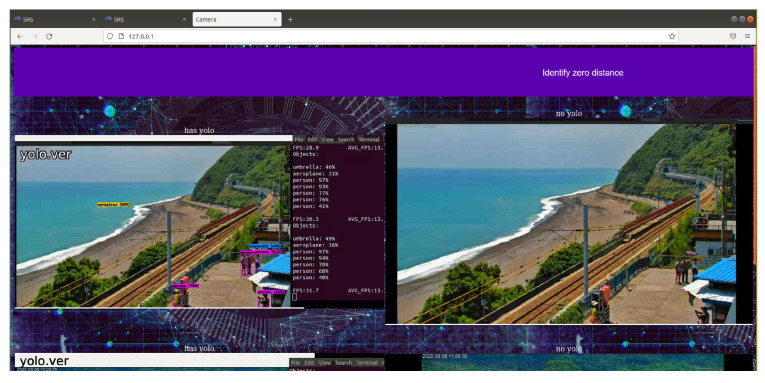
Real-time image recognition of Taitung Duoliang Station.

**Figure 21:**
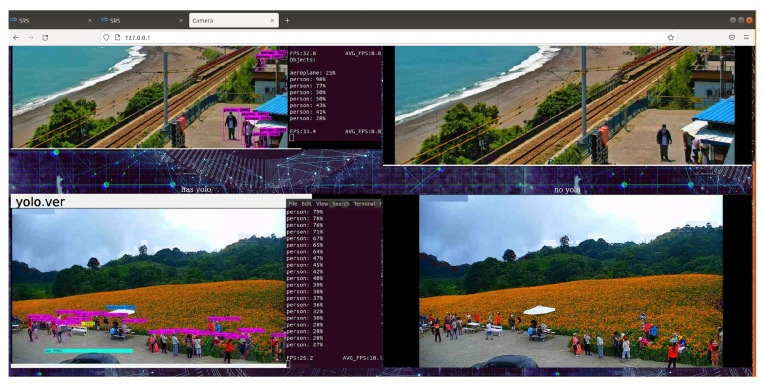
Real-time image recognition of Chike Mountain in Hualien.

The use of the Taitung Duoliang Station live video (Figure 19 and Figure 20—top) and Hualian Chike Mountain real-time video (Figure 21—bottom) were, respectively, provided by Taitung County Government Transportation and Tourism Development Office and Huadong Zong. Taking the YouTube real-time video live broadcast built by the National Scenic Management Office of the Valley as an example, and importing OBS (Open Broadcaster Software), we can stream out a stable signal for identification (Figure 19).

### 5.3. Specific Target

**Figure 22:**
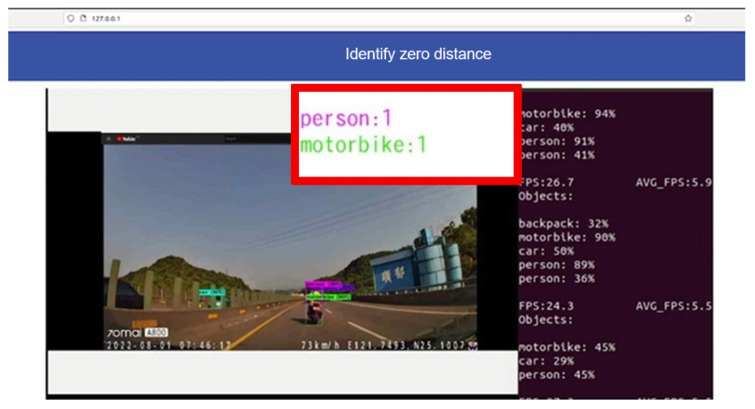
System overview 2 (The event of a locomotive running into a national highway by mistake).

We can carry out recognition training for specific targets, such as masks and drink cups, and when the training accuracy reaches more than 85%, it can be applied to the system. When specific information appears, prompts and warnings will pop up immediately, such as those shown in Figure 22, where a locomotive is accidentally crossing a national highway. When a locomotive appears in the image recognition data, the system immediately shows a warning.

### 5.4. System Results Overview

**Figure 23:**
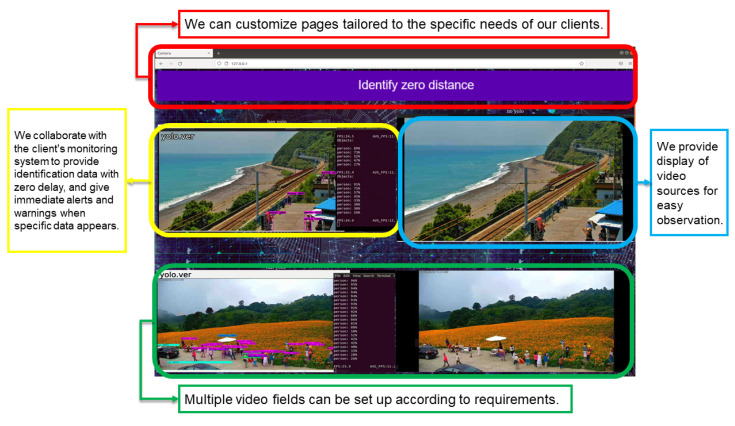
System overview 1 (using Taitung Duoliang Station live video (above) and real-time video of Chike Mountain in Hualien (pictured below) for the real-time management of the flow of people).

The overall system interface is as follows (Figure 23). In addition to the functional design of the development concept, the design of the user interface can be flexibly adjusted according to requirements.

Figure 22 is an example of using WoWtchout—a map-based driving image sharing platform on the 11.1 km west of Taiwan Line 62. The goal is to ascertain whether the simulation system can identify the difference between the vehicle types on the road and cooperate with the special layout for viewing the identified screen. With a simple design, it can be clearly viewed to achieve the monitoring effect.

### 5.5. Energy-Constrained Environments

In energy-constrained environments, analyzing system power consumption is crucial, particularly when utilizing various streaming media protocols. The choice of protocol can significantly impact the overall energy efficiency of edge computing devices, such as those using the NVIDIA Jetson Xavier NX. WebRTC, known for its low latency capabilities, typically achieves delays of around 5 s, making it suitable for real-time applications. However, this protocol demands higher CPU usage, often exceeding 40% during operation, which can lead to increased power consumption. This higher resource requirement may pose challenges in energy-constrained settings, where battery life and energy efficiency are critical. In contrast, RTMP (Real-Time Messaging Protocol) generally exhibits lower power consumption compared to WebRTC. RTMP is designed for streaming audio, video, and data over the Internet, and its processing requirements are less intensive, making it a more energy-efficient option. This feature proves especially advantageous when processing multiple streams concurrently, as it can effectively reduce the overall power consumption. HTTP Live Streaming (HLS) also presents unique challenges in energy-constrained environments. While HLS is compatible with firewalls and proxies, it typically experiences higher latency, often exceeding 10 s. HLS’s reliance on HTTP connections influences its power consumption, potentially increasing overhead and energy consumption during streaming sessions. The need for continuous connections can further contribute to higher power consumption compared to RTMP. The comparative analysis of power consumption across these protocols reveals that while WebRTC offers low latency, its higher power consumption may not be suitable for all scenarios. Conversely, RTMP and HLS provide lower power consumption, making them viable alternatives for applications requiring energy efficiency. The findings indicate that the system can support multiple simultaneous connections, but performance degrades significantly with more than three devices, leading to increased power usage. This limitation highlights the importance of optimizing both the chosen streaming protocol and the overall system architecture to minimize energy consumption.

### 5.6. System Security

This section involves an analysis of system security in edge computing environments. Security is a critical concern in edge computing environments, where devices often operate in less controlled settings compared to traditional data centers. While the integration of EI in 5G networks enhances data processing capabilities, it also introduces vulnerabilities that require attention to safeguard sensitive information. The following are methods to protect information: Data Protection Measures: To safeguard data in edge computing, it is essential to implement robust encryption protocols for data in transit and at rest. Utilizing secure communication protocols, such as HTTPS and TLS, can help protect data transmitted between edge devices and servers. Additionally, employing end-to-end encryption ensures that data remain secure from unauthorized access during transmission, which is particularly important for applications involving real-time video processing and streaming.

Privacy Measures: Edge computing systems must also prioritize privacy measures. Implementing access control mechanisms can restrict data access to authorized users only, thereby minimizing the risk of data breaches. Furthermore, anonymizing sensitive data before processing not only protects user privacy but also enables the extraction of valuable insights from the data. Challenges and considerations: Despite these measures, challenges remain in ensuring comprehensive security in edge computing environments. The distributed nature of edge devices can complicate security management, as each device may have different security requirements and vulnerabilities. Additionally, the reliance on third-party services and cloud integration can introduce additional risks, necessitating thorough vetting of service providers and their security practices. In conclusion, while edge computing offers significant advantages in terms of performance and efficiency, it is imperative to address security concerns through effective data protection and privacy measures. Continuous monitoring and updating of security protocols will be essential to adapt to evolving threats in this dynamic environment.

### 5.7. Real-World Applications

The streaming service application, built using DeepStream and Simple Realtime Server on the NVIDIA Jetson Xavier NX, has several practical applications, particularly in real-time video processing and surveillance systems. For instance, traffic monitoring can utilize this system, which analyzes video feeds to detect and classify vehicles, thereby providing real-time data to traffic management centers. DeepStream’s efficient video processing features enhance this capability by enabling rapid object detection and tracking. In real-world scenarios, the system has demonstrated its effectiveness in environments such as public transportation hubs and urban areas, where it can monitor foot traffic and vehicle movement. The integration of OBS enables stable signal streaming for identification purposes, as shown in live video examples from Taitung Duoliang Station and Hualian Chike Mountain. This application not only improves situational awareness but also aids in decision-making processes for urban planning and emergency response. However, there are challenges associated with deploying this system in practical applications. Observing performance degradation when multiple devices connect simultaneously to the Simple Realtime Server is a significant issue. The load testing results indicated that while the system could support up to 15 simultaneous connections, performance significantly declined with more than three devices, leading to increased latency and connection failures. This limitation highlights the need for further optimization and potentially more robust hardware solutions to enhance scalability and reliability in high-demand environments. Moreover, the reliance on WebRTC for low-latency streaming introduces additional challenges, such as network penetration issues and higher CPU usage, which can exceed 40% during operation. These factors must be carefully managed to ensure that the system remains efficient and effective in real-world applications, particularly in scenarios requiring real-time data processing and analysis. Overall, while the constructed system shows outstanding promise, addressing these challenges will be crucial for its successful deployment in various practical contexts.

### 5.8. Future Directions

Further directions for the research can focus on several key areas to enhance the effectiveness and scalability of the streaming service application built with DeepStream and Simple Realtime Server.

1. Scalability Improvements: Given the findings that performance degrades significantly with more than three simultaneous connections, future research should explore optimization techniques to enhance scalability. This could involve investigating load balancing strategies or implementing more robust hardware solutions to support a higher number of concurrent users without compromising performance.
2. Protocol Optimization: While WebRTC has demonstrated low latency capabilities, its high CPU usage (often exceeding 40%) poses challenges for devices with limited processing power. Future studies could focus on optimizing WebRTC’s performance or exploring alternative protocols that maintain low latency while reducing resource consumption, such as enhancing RTMP or HLS implementations.
3. Integration with AI Technologies: The integration of AI technologies, such as advanced object detection and recognition algorithms, could further enhance the capabilities of the streaming service. Research could investigate the application of more sophisticated models, like YOLOv4, to improve accuracy and speed in real-time video analytics.
4. Security Enhancements: As security is crucial in edge computing environments, future work should address potential vulnerabilities associated with the streaming protocols and the overall system architecture. Implementing robust encryption and access control measures will be essential to protect sensitive data and ensure user privacy.
5. Cross-Platform Compatibility Testing: Although the current research highlights WebRTC’s cross-platform support, further testing across a wider range of devices and operating systems could provide insights into compatibility issues and performance variations. This would help to ensure a seamless user experience across different platforms. By addressing these areas, future research can significantly contribute to the development of more efficient, secure, and scalable streaming service applications in edge computing environments.

## 6. Conclusions

This paper successfully integrated DeepStream and Simple Realtime Server using Docker and Docker Compose on the NVIDIA Jetson Xavier NX, demonstrating that containerization does not compromise performance compared to traditional physical machines. The experimental results confirmed that WebRTC provides superior real-time streaming capabilities, achieving lower latency compared to HLS and RTMP, with delays around 5 s for WebRTC versus over 10 s for HLS. Additionally, the load testing revealed that while the system can support multiple simultaneous connections, performance significantly degrades with more than three devices, indicating the limitations of the current hardware setup. The findings contribute valuable insights into the development of efficient edge computing architectures for real-time video processing and streaming applications, paving the way for future research and enhancements in this domain. Furthermore, the successful implementation of this architecture highlights the potential of using Docker for scalable and efficient deployment in edge computing environments.

## Figures and Tables

**Table 1:** WebRTC cross platform support.

Device	Chrome	Firefox	Microsoft Edge	Safari
Windows 10	O	O	O	
Android Samsung Note8	O	O	O	
iPhone 12	O	O	O	O
iPad	O	O		O
Macbook	O	O		O

**Table 2:** System device specifications.

Component	Specifications
GPU	384-core NVIDIA Volta™ GPU with 48 Tensor Cores
CPU	6-core NVIDIA Carmel ARM®v8.2 64-bit CPU 6 MB L2 + 4 MB L3
Storage	16 GB eMMC 5.1
Power	10 W | 15 W
PCIe	1 × 1 + 1 × 4 (PCIe Gen3, Root Port & Endpoint)
Video Encode	2× 464 MP/s (HEVC) 2× 4K @ 30 (HEVC) 6× 1080p @ 60 (HEVC) 14× 1080p @ 30 (HEVC)
Video Decode	2× 690 MP/s (HEVC) 2× 4K @ 60 (HEVC) 4× 4K @ 30 (HEVC) 12× 1080p @ 60 (HEVC) 32× 1080p @ 30 (HEVC) 16× 1080p @ 30 (H.264)
Display	2 multi-mode DP 1.4/eDP 1.4/HDMI 2.0
DL Accelerator	2× NVDLA Engines
Vision Accelerator	7-Way VLIW Vision Processor
Networking	10/100/1000 BASE-T Ethernet
Mechanical	45 mm × 69.6 mm 260-pin SO-DIMM connector

**Table 3:** Software version.

Software	Version
Ubuntu	18.04.5
DeepStreamSDK	5.1.0
Cuda	10.2
TensorRT	7.1
cuDNN	8.0
libNVWarp360	2.0.1d3
gstreamer	1.0
Docker	19.03.6
Docker Compose	1.29.1
Python	3.6
Simple Realtime Server	4.0
Apache	2.4.29
ffmpeg	3.4.8-0ubuntu0.2
OBS	27.2.4 (Linux)
YOLOV4	2020

**Table 4:** SRS Summary.

SRS/6.0.33		OS System		10 Load		Others	
Alive	4d 23:08:29	Alive	4d 04:59:04	Internet	0 Kbps/0 Kbps	CPU	16/16
CPU	3.00%/1600.00%	CPU	448.00%/1600.00%	Intranet	28 Mbps/25 Mbps	PID	12086
Memory	2% 502 MB/31 GB	Memory	30% 9 GB/31 GB	Conns	76 30 21 13	PPID	10741
Network	4/8 Mbps/3 Mbps	Load	4.73/4.61/4.51	Disk	0% OKBps 4 MBps	Ready	Yes

**Table 5:** Speed and accuracy.

FPS	Motorbike	Car	Person
24.5	93%; 92%; 90%; 89%; 87%; 81%	87%; 86%	96%; 90%; 90%; 87%
28.4	93%; 91%; 90%; 89%; 86%	87%; 86%	96%; 90%; 90%; 87%
27.2	93%; 92%; 90%; 89%; 86%	87%; 86%	96%; 90%; 90%; 87%
29.4	93%; 92%; 90%; 89%; 86%	87%; 86%	96%; 90%; 90%; 87%
28.7	93%; 92%; 90%; 89%; 86%	87%; 86%	96%; 90%; 90%; 87%

## Data Availability

Data available on request due to restrictions in accordance with our confidentiality policy.

## References

[1] Sittón-Candanedo I., Alonso R.S., Corchado J.M., Rodríguez-González S., Casado-Vara R. (2019). A review of edge computing reference architectures and a new global edge proposal. Future Gener. Comput. Syst..

[2] Ning Z., Huang J., Wang X., Rodrigues J.J., Guo L. (2019). Mobile edge computing-enabled Internet of vehicles: Toward energy-efficient scheduling. IEEE Netw..

[3] Chen J., Li K., Deng Q., Li K., Philip S.Y. (2019). Distributed deep learning model for intelligent video surveillance systems with edge computing. IEEE Trans. Ind. Inform..

[4] Islam A., Debnath A., Ghose M., Chakraborty S. (2021). A survey on task offloading in multi-access edge computing. J. Syst. Archit..

[5] Furano G., Meoni G., Dunne A., Moloney D., Ferlet-Cavrois V., Tavoularis A., Byrne J., Buckley L., Psarakis M., Voss K.O. (2020). Towards the use of artificial intelligence on the edge in space systems: Challenges and opportunities. IEEE Aerosp. Electron. Syst. Mag..

[6] Wan S., Ding S., Chen C. (2022). Edge computing enabled video segmentation for real-time traffic monitoring in internet of vehicles. Pattern Recognit..

[7] Potdar A.M., Narayan D., Kengond S., Mulla M.M. (2020). Performance evaluation of docker container and virtual machine. Procedia Comput. Sci..

[8] Masdari M., Nabavi S.S., Ahmadi V. (2016). An overview of virtual machine placement schemes in cloud computing. J. Netw. Comput. Appl..

[9] Ali A.A., El-Kalioby M., Abouelhoda M. (2016). The case for Docker in multicloud enabled bioinformatics applications. Bioinformatics and Biomedical Engineering, Proceedings of the 4th International Conference, IWBBIO 2016, Granada, Spain, 20–22 April 2016, Proceedings 4.

[10] Guidotti R., Soldani J., Neri D., Brogi A. (2018). Explaining successful docker images using pattern mining analysis. Software Technologies: Applications and Foundations, Proceedings of the STAF 2018 Collocated Workshops, Toulouse, France, 25–29 June 2018, Revised Selected Papers.

[11] Cito J., Ferme V., Gall H.C. (2016). Using docker containers to improve reproducibility in software and web engineering research. Proceedings of the ACM 38th International Conference on Software Engineering Companion (ICSE-C).

[12] Kumar P., Shah M. (2020). To Build Scalable and Portable Blockchain Application Using Docker. Soft Computing: Theories and Applications.

[13] Qiu M. (2021). Smart Computing and Communication: 5th International Conference, SmartCom 2020, Paris, France, 29–31 December 2020, Proceedings.

[14] Abdulghafoor N.H., Abdullah H.N. (2021). Real-time moving objects detection and tracking using deep-stream technology. J. Eng. Sci. Technol..

[15] Simple Realtime Server (SRS). https://github.com/ossrs/srs.

[16] RTMP. https://helpx.adobe.com/adobe-media-server/dev/stream-on-demand-media-rtmp.html.

[17] Durak K., Akcay M.N., Erinc Y.K., Pekel B., Begen A.C. (2020). Evaluating the performance of apple’s low-latency HLS. Proceedings of the 2020 IEEE 22nd International Workshop on Multimedia Signal Processing (MMSP).

[18] HTTP Live Streaming. https://zh.wikipedia.org/wiki/HTTP_Live_Streaming.

[19] Chodorek A., Chodorek R.R., Sitek P. (2021). UAV-based and WebRTC-based open universal framework to monitor urban and industrial areas. Sensors.

[20] Chodorek A., Chodorek R.R., Yastrebov A. (2022). The Prototype Monitoring System for Pollution Sensing and Online Visualization with the Use of a UAV and a WebRTC-Based Platform. Sensors.

[21] García B., Gallego M., Gortázar F., Bertolino A. (2019). Understanding and estimating quality of experience in WebRTC applications. Computing.

[22] Hu X., Liu Y., Zhao Z., Liu J., Yang X., Sun C., Chen S., Li B., Zhou C. (2021). Real-time detection of uneaten feed pellets in underwater images for aquaculture using an improved YOLO-V4 network. Comput. Electron. Agric..

[23] Ullah S., Kim D.H. (2020). Benchmarking Jetson platform for 3D point-cloud and hyper-spectral image classification. Proceedings of the 2020 IEEE International Conference on Big Data and Smart Computing (BigComp).

[24] Feng H., Mu G., Zhong S., Zhang P., Yuan T. (2022). Benchmark analysis of yolo performance on edge intelligence devices. Cryptography.

[25] Zhu J., Feng H., Zhong S., Yuan T. (2022). Performance analysis of real-time object detection on Jetson device. Proceedings of the 2022 IEEE/ACIS 22nd International Conference on Computer and Information Science (ICIS).

[26] Grzesik P., Mrozek D. (2021). Metagenomic analysis at the edge with Jetson Xavier NX. Proceedings of the International Conference on Computational Science.

[27] Carofiglio G., Muscariello L., Augé J., Papalini M., Sardara M., Compagno A. Enabling icn in the internet protocol: Analysis and evaluation of the hybrid-icn architecture. Proceedings of the 6th ACM Conference on Information-Centric Networking.

[28] Dai Y., Xu D., Maharjan S., Qiao G., Zhang Y. (2019). Artificial intelligence empowered edge computing and caching for internet of vehicles. IEEE Wirel. Commun..

[29] Xu X., Xu A., Jiang Y., Wang Z., Wang Q., Zhang Y., Wen H. (2020). Research on Security Issues of Docker and Container Monitoring System in Edge Computing System. Journal of Physics: Conference Series.

[30] Jansen C., Witt M., Krefting D. (2016). Employing docker swarm on openstack for biomedical analysis. Proceedings of the International Conference on Computational Science and Its Applications.

[31] Sharma I., Manohit, Bhandari A. (2021). Implementing Slowloris DoS Using Docker. Advances in Information Communication Technology and Computing.

[32] Brogi A., Pahl C., Soldani J. (2018). On enhancing the orchestration of multi-container Docker applications. Proceedings of the European Conference on Service-Oriented and Cloud Computing.

[33] Nguyen T.L., Nou R., Lebre A. (2019). YOLO: Speeding up VM and Docker Boot Time by reducing I/O operations. Proceedings of the European Conference on Parallel Processing.

[34] Saxena D., Sharma N. (2021). Analysis of Docker Performance in Cloud Environment. Advances in Information Communication Technology and Computing.

[35] Morabito R., Farris I., Iera A., Taleb T. (2017). Evaluating performance of containerized IoT services for clustered devices at the network edge. IEEE Internet Things J..

[36] Qiao Y., Shen S., Zhang C., Wang W., Qiu T., Wang X. (2024). EdgeOptimizer: A programmable containerized scheduler of time-critical tasks in Kubernetes-based edge-cloud clusters. Future Gener. Comput. Syst..

[37] Oleghe O. (2021). Container placement and migration in edge computing: Concept and scheduling models. IEEE Access.

[38] List M. (2017). Using docker compose for the simple deployment of an integrated drug target screening platform. J. Integr. Bioinform..

[39] Pentyala S.K. (2017). Emergency communication system with Docker containers, OSM and Rsync. Proceedings of the 2017 International Conference On Smart Technologies For Smart Nation (SmartTechCon).

